# Identification of Novel *PTPRQ* and *MYO1A* Mutations in An Iranian Pedigree with Autosomal Recessive Hearing Loss

**DOI:** 10.22074/cellj.2018.4805

**Published:** 2018-01-01

**Authors:** Farah Talebi, Farideh Ghanbari Mardasi, Javad Mohammadi Asl, Saeed Tizno, Marziye Najafvand Zadeh

**Affiliations:** 1 Ahvaz Welfare Organization, Ahvaz, Iran; 2 Department of Nursing, Shoushtar Faculty of Medical Sciences, Shoushtar, Iran; 3 Department of Medical Genetics, Faculty of Medicine, Ahvaz Jundishapur University of Medical Sciences, Ahvaz, Iran; 4 Department of ENT, Faculty of Medicine, Guilan University of Medical Sciences, Guilan, Iran

**Keywords:** Hearing Loss, *MYO1A*, Novel Variant, *PTPRQ*

## Abstract

Autosomal recessive non-syndromic hearing loss (ARNSHL) is defined as a genetically heterogeneous disorder. The aim of the present study was to screen for pathogenic variants in an Iranian pedigree with ARNSHL. Next-generation targeted sequencing of 127 deafness genes in the proband detected two novel variants, a homozygous missense variant in *PTPRQ* (c.2599 T>C, p.Ser867Pro and a heterozygous missense variant in *MYO1A* (c.2804 T>C, p.Ile935Thr), both of which were absent in unaffected sibs and two hundred unaffected controls. Our results suggest that the homozygous *PTPRQ* variant maybe the pathogenic variant for ARNSHL due to the recessive nature of the disorder. Nevertheless, the heterozygous *MYO1A* may also be involved in this disorder due to the multigenic pattern of ARNSHL. Our data extend the mutation spectrum of *PTPRQ* and *MYO1A*, and have important implications for genetic counseling in unaffected sibs of this family. In addition, *PTPRQ* and *MYO1A* pathogenic variants have not to date been reported in the Iranian population.

## Introduction

Hearing impairment is one of the most common *sensorineural* disorders in humans, affecting approximately one in 500-1,000 newborns. Hereditary hearing impairments are mainly transmitted in an autosomal dominant or recessive fashion (1) with mitochondrial (2) or X-linked (3) inheritance reported in frequently. Considering the isolated forms, about 80% of hereditary deafness cases manifest as autosomal recessive non-syndromic hearing loss (ARNSHL) (4). To date, more than 100 genes have been implicated in ARNSHL (5).

Molecular diagnosis plays a key role in clinical management, prognosis evaluation and prenatal diagnosis (PND) for ARNSHL families (6). However, the genetic heterogeneity of hearing impairment had undermined genetic diagnosis in most cases until recently. With the advent of next-generation sequencing (NGS) technology, heterogeneous disorders are now open to routine genetic testing and comprehensive genetic analysis. Targeted NGS of the identified deafness genes (a “gene panel” that generally covers the exons and flanking intronic sequences) can provide a basis for a broad first-step study of pathogenic variants in ARNSHL (7). We thus aimed to screen the deafness gene panel in a proband with ARNSHL and of Iranian origin. Herein, we report two novel missense pathogenic variants in *PTPRQ* and *MYO1A*, both of which may explain the ARNSHL phenotype in the proband. 

## Case report

**Fig.1:**
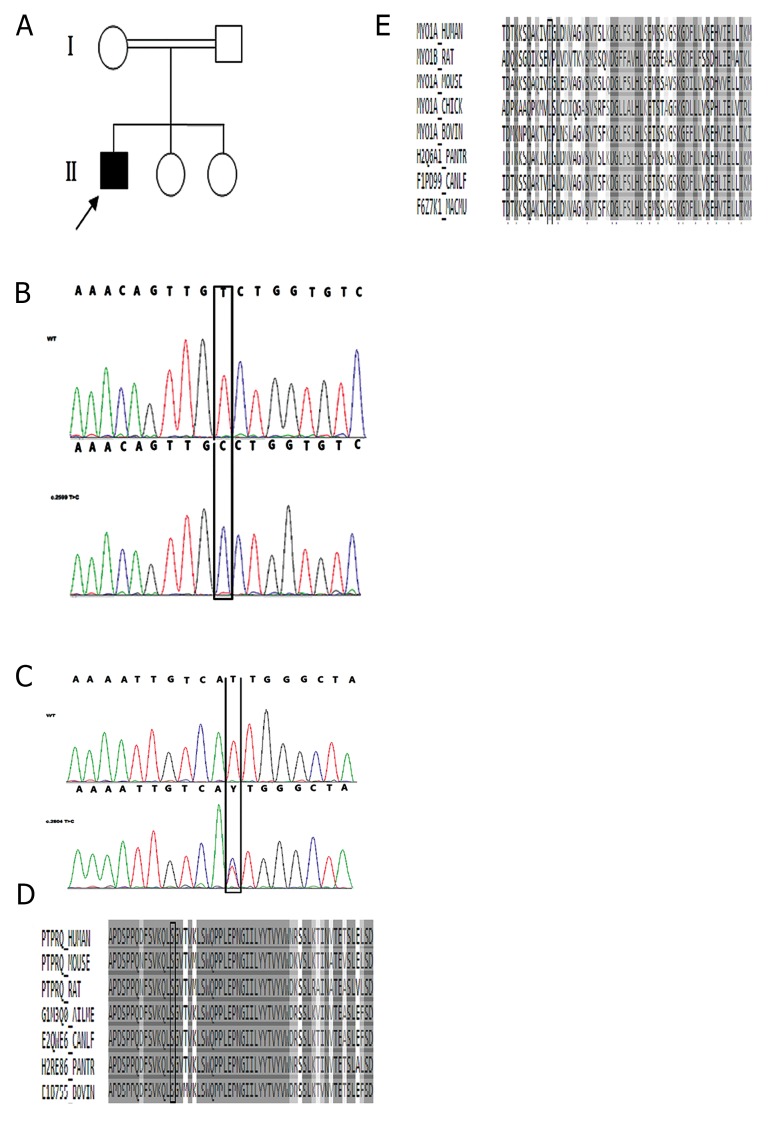
Genetic analysis of the ARNSHL proband. A. Pedigree of family B with ARNSHL, the proband is denoted in black. Partial sequences of B. *PTPRQ*, C. *MYO1A* in the proband showing that homozygous mutation (c.2599T>C) in *PTPRQ* and the heterozygous mutation (c.2804 T>C) in *MYO1A*, both co-segregating with the phenotype. Mutated nucleotides are marked with vertical lines (black). Protein alignment shows conservation of residue D. 867 in *PTPRQ*, and E.935 in *MYO1A* across seven and eight species respectively. These two novel mutations occur at evolutionarily conserved amino acid positions marked with vertical lines (black).

The proband is a 23-year-old Iranian male with a clinical diagnosis of hearing impairment (Fig .1A). No exact complications have been reported during his perinatal period. However, at age of 21 months, his mother suspected hearing loss because of his poor response to sound. He was born from a consanguineous marriage (first cousin unaffected parents). There was no family history of inherited diseases such as ARNSHL or congenital malformations in his pedigree. Two hundred unrelated subjects of Iranian origin with normal hearing were screened for the pathogenic variants as controls. Written informed consent was obtained from all participants according to the guidelines of the Ethics Committee of the Ministry of Health and Medical Education of Iran.

Blood samples were collected from the proband and his parents. Genomic DNA was extracted from blood samples of all participants using the standard salting out method (8). Targeted NGS was carried out by using a custom designed NimbleGen chip capturing 127 hearing impairment genes including but not limited to *PTPRQ, GJB6, MYO1A, MYO7A, SLC26A4,* and *MTRNR1* (BGI-Clinical Laboratories, China). The genomic region containing the variant were amplified (primer sequences are available upon request) in 25 µL volumes and 35 cycles: 95°C for one minute, 65°C for 40 seconds and 72°C for one minute and then the polymerase chain reaction (PCR) product was sequenced with direct sanger sequencing carried out with automated DNA sequencer (ABI3130, Applied Biosystems, USA) (validation with a second independent sample of DNA) to confirm presence of potential pathogenic variants in the proband and his parents for segregation analysis.

The frequency of the detected variants was checked in the 1000 genomes database (http://WWW.1000genomes. org/.). Next, in silico functional prediction of the missense variants were performed with bioinformatics tools including Sorting Intolerant from Tolerant (SIFT) (9), Polymorphism Phenotyping V2 (PolyPhen2) (10) and Mutation Taster (11).

**Fig.2:**
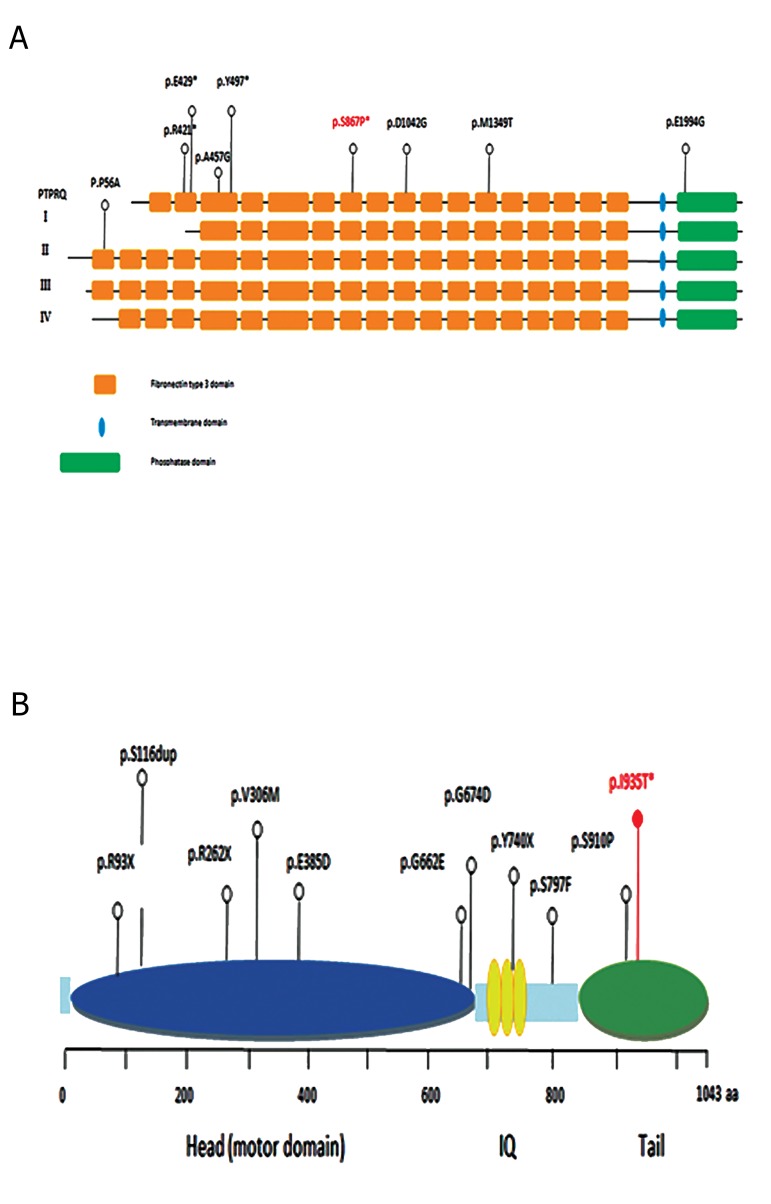
Diagram structure of *PTPRQ* and Myosin-IA proteins. Schematic of A. *PTPRQ* and B. Myosin-IA proteins show the locations of the pathogenic variants in humans. The two novel mutations reported in this study are shown in red font (p.Ser867Pro and p.Ile935Thr).

All genomic data analysis including read alignment, variant calling and novel mutation identification was undertaken by BGI that detected two novel variants in *PTPRQ* and *MYO1A* co-segregating in the family. The variant in exon 17 of *PTPRQ* (c.2599T>C) results in a serine to proline substitution at codon 867 (Ser867Pro) (Fig .1B). The second variant was found in exon 26 of *MYO1A* (c.2804 T>C) (Fig .1C), leading to an isoleucine to threonine substitution (Ile935Thr). Both missense variants were predicted to be pathogenicity the three prediction tools (Table 1). Reported mutations in *PTPRQ* and *MYO1A* are summarized in Table 2, and 3 respectively. Interestingly, no pathogenic variants were identified in the other 125 genes in the proband. The two detected variants were confirmed by sanger sequencing. Both missense variants alter highly evolutionary conserved amino acids (Fig .1D, E). To confirm pathogenicity, presence of the two variants was checked in unaffected individuals in the pedigree. The unaffected parents and one of his sisters (II-3) were heterozygous for the *PTPRQ* variant while the *MYO1A* variant was only identified in the mother in a heterozygous state. Both variants were not detected in the other sister (II-2) and the 200 healthy controls of Iranian origin. Figure 2 shows the locations of these variants. 

**Table 1:** Results of in silico prediction tools for functional effect of the novel missense mutations


Gene/Variant	SIFT score	PolyPhen score	Mutation taster

ENST00000614701, S867P		0.787 (possibly damaging)	disease causing
ENST00000300119, I935T		0.908 (possibly damaging)	disease causing


**Table 2:** Reported mutations in PTPRQ


Origin	Pathogenic variant	Protein effect	Domain	Exon	Type of mutation	Inheritance pattern	Zygosity

**Palestinian**	c.1285C>T	p.Gln429Stop	EC	9	Nonsense	AR	Homozygous
**Dutch**	c.1491T>A	p.Tyr497Stop	EC	10	Nonsense	AR	Homozygous
**Moroccan**	c.1369A>G	p.Ala457Gly	EC	10	Missense	AR	Homozygous
**Chinese**	c.3125A>G	p.Asp1042Gly	EC	20	Missense	AR	Homozygous
**Chinese**	c.5981A>G	p.Glu1994Gly	EC	37	Missense	AR	Homozygous
**Japanese**	c.166C>G	p.Pro56Ala	EC	2	Missense	AR	Compoundheterozygous
**Japanese**	c.1261C>T	p.Arg421Stop	EC	9	Nonsense	AR	Homozygous
**Japanese**	c.4046T>C	p.Met1349Thr	EC	25	Missense	AR	Compoundheterozygous
**Japanese**	c.6453+3delA	-	CP	41	Splice site	AR	Compoundheterozygous
**Iranian**	c.2599T>C	Ser867Pro	EC	17	Missense	AR	Homozygous


CP; Cytoplasmic domain, EC; Extracellular domain, and AR; Autosomal recessive.

**Table 3:** Reported mutations in MYO1A


Origin	Pathogenic variant	Protein effect	Exon	Domain	Type of mutation	Inheritance pattern	Zygosity

**Italian**	277C/T	R93X	3	Myosin motor	Nonsence	AD	Heterozygous
**Italian**	349-350A	349-350insCTT	4	Myosin motor	Insertion	AD	Heterozygous
**Italian**	916G/A	V306M	10	Myosin motor	Missence	AD	Heterozygous
**Italian**	1155G/T	E385D	12	Myosin motor	Missence	AD	Heterozygous
**Italian**	1985G/A	G662E	18	Myosin motor	Missence	AD	Heterozygous
**Italian **	2021G/A	G674D	18	Myosin motor	Missence	AD	Heterozygous
**Italian**	2390C/T	S797F	22	-	Missence	AD	Heterozygous
**Italian**	2728T/C	S910P	25	TH1	Missence	AD	Heterozygous
**Pakistani**	c.784C>T	p.Arg262∗	10	Myosin motor	nonsense	AD	Heterozygous
**German**	c.2220T>G	p.Tyr740∗	21	IQ 2	nonsense	AD	Heterozygous
**Iranian**	c.2804T>C	I935T	26	TH1	Missence	AR/ compound heterozygous	Heterozygous


TH1; Class I myosin tail homology, AD; Autosomal dominant, and AR; Autosomal recessive.

## Conclusion

Here we report two novel missense variants in *PTPRQ* and *MYO1A* in an Iranian family displaying hearing loss. Protein Tyrosine Phosphatase, Receptor Type Q (*PTPRQ*) is a stereociliar membrane protein, composed of three domains which include an extracellular domain (containing 18 fibronectin III repeats), a membrane spanning domain (trans membrane domain) and a cytoplasmic domain (phosphatase domain) (12-14). It plays key roles in cell shape changes, regulation of actin filament organization and formation of stereocilia in hair cells of the inner ear (15) with its loss or malfunction resulting in shaft connector malformation of hair cell stereocilia (16). 

The novel homozygous *PTPRQ* variant detected in the proband is located in the *fibronectin type III-9* domain (extracellular domain). This extracellular domain is able to bind ligands including extracellular proteins, collagen and heparin as well as ligands on the cell (17-19). The wild-type residue is polar while the mutant residue is non-polar, thus likely to affect *PTPRQ* interactions with ligands. 

Additionally, this is the first *PTPRQ* variant found in an Iranian population. To date, 9 variants in *PTPRQ* have been reported. All *PTPRQ* variants previously reported were detected in prelinguistic or congenital hearing loss patients (20). The proband in this study had congenital hearing loss, consistent with previous reports. Of the 9 reported *PTPRQ* variants, five were missense variants in the extracellular (EC) domain of which three were found in a homozygous state [p.A457G in Morocco (12), and p.D1042G and p.E1994G in China (21)] and two in a heterozygous state [p.P56A and p.M1349T in Japan (20)]. Three were also nonsense variants in the EC domain that were found in homozygous [p.Q429X in Palestine (13) and p.Y497X in Holland (112)] or heterozygous [p.R421X in Japan (20)] state. The ninth variant was a heterozygous splice site variant (c.6453+3delA) detected in a Japanese family (20). 

We also identified a novel heterozygous variant in *MYO1A* as a potentially causative variant of congenital ARNSHL in the proband. *MYO1A* encodes Myosin- IA, a protein with 1043 amino acids, belonging to the myosin super family (22, 23). MYO1A contains three core domains, an N-terminal motor domain, a central neck region made up of IQ motifs and a tail region. MYO1A functions as an actin-based molecular motor and is implicated in directing the movement of organelles along the actin filaments (24). 

Variants within this gene have been reported to cause ARNSHL (25). To date, 10 recessive variants in *MYO1A* have been shown to be associated with ARNSHL in patients of Italian, German and Pakistani descent. However, variants in *MYO1A* have not to date been reported in the Iranian population. 

The c.2804 T>C variant located in the *C-terminal tail homology-1 (TH1) domain, which is responsible for membrane* binding (26). Therefore, missense variants that alter a nonpolar aliphatic amino acid to polar amino acids with a hydroxyl group may modify the interaction of the tail domain with membranous compartments and alter its movement. Therefore this novel variant is likely to negatively affect the function of the TH1 domain. ARNSHL has an autosomal recessive inheritance pattern and since neither parents nor the proband are homozygous, it is unlikely to be causal in this case. However, this variant might cause pathogenicity in case another variant is acquired in future generations and result in compound heterozygosity. 

Our findings confirm that two novel variants in *PTPRQ* and *MYO1A* may be causative of ARNSHL in a consanguineous Iranian family. In conclusion, by using NGS in this study, we show that this method can be useful for detecting rare causative genetic variants in ARNSHL patients, such as those detected in *MYO1A* and *PTPRQ*.
